# Stress hyperglycemia ratio and in-hospital prognosis in non-surgical patients with heart failure and type 2 diabetes

**DOI:** 10.1186/s12933-022-01728-w

**Published:** 2022-12-26

**Authors:** Yiling Zhou, Li Liu, Hongmei Huang, Nan Li, Jidong He, Heling Yao, Xiaochi Tang, Xiangyang Chen, Shengzhao Zhang, Qingyang Shi, Furong Qu, Si Wang, Miye Wang, Chi Shu, Yuping Zeng, Haoming Tian, Ye Zhu, Baihai Su, Sheyu Li, The WECODe Study Group

**Affiliations:** 1 Department of Endocrinology and Metabolism, West China Hospital, Sichuan University, Chengdu, 610041 China; 2 Department of Endocrinology and Metabolism, Second People’s Hospital of Ya’an City, Ya’an, 625000 China; 3 Department of Endocrinology and Metabolism, The First People’s Hospital of Shuangliu District, Chengdu, 610200 China; 4 The Informatic Center, West China Hospital, Sichuan University, Chengdu, 610041 China; 5 Department of Pharmacy, Karamay Central Hospital, Karamay, 834000 China; 6 Department of Pharmacy, West China Hospital, Sichuan University, Chengdu, 610041 China; 7 Chinese Evidence-Based Medicine Center, Cochrane China Center and MAGIC China Center, West China Hospital, Sichuan University, Chengdu, 610041 China; 8 Department of General Practice, West China Hospital, Sichuan University, Chengdu, 610041 China; 9 Department of Cardiology, West China Hospital, Sichuan University, Chengdu, 610041 China; 10 Department of Vascular Surgery, West China Hospital, Sichuan University, Chengdu, 610041 China; 11 Department of Laboratory Medicine, West China Hospital, Sichuan University, Chengdu, 610041 China; 12 Department of Nephrology, West China Hospital, Sichuan University, Chengdu, 610041 China

**Keywords:** Heart failure, Type 2 diabetes, Stress hyperglycemia, Acute kidney injury, Death, Cardiovascular events, Hospitalization

## Abstract

**Objective:**

To evaluate the impact of stress hyperglycemia on the in-hospital prognosis in non-surgical patients with heart failure and type 2 diabetes.

**Research design and methods:**

We identified non-surgical hospitalized patients with heart failure and type 2 diabetes from a large electronic medical record-based database of diabetes in China (WECODe) from 2011 to 2019. We estimated stress hyperglycemia using the stress hyperglycemia ratio (SHR) and its equation, say admission blood glucose/[(28.7 × HbA1c)− 46.7]. The primary outcomes included the composite cardiac events (combination of death during hospitalization, requiring cardiopulmonary resuscitation, cardiogenic shock, and the new episode of acute heart failure during hospitalization), major acute kidney injury (AKI stage 2 or 3), and major systemic infection.

**Results:**

Of 2875 eligible Chinese adults, SHR showed U-shaped associations with composite cardiac events, major AKI, and major systemic infection. People with SHR in the third tertile (vs those with SHR in the second tertile) presented higher risks of composite cardiac events ([odds ratio, 95% confidence interval] 1.89, 1.26 to 2.87) and major AKI (1.86, 1.01 to 3.54). In patients with impaired kidney function at baseline, both SHR in the first and third tertiles anticipated higher risks of major AKI and major systemic infection.

**Conclusions:**

Both high and low SHR indicates poor prognosis during hospitalization in non-surgical patients with heart failure and type 2 diabetes.

**Supplementary Information:**

The online version contains supplementary material available at 10.1186/s12933-022-01728-w.

## Highlights

- Why did we undertake this study?
  Stress hyperglycemia is classic but a long-overlooked pathophysiological process and a recently validated parameter, stress hyperglycemia ratio (SHR), facilitates its estimation.

(b) What is the specific question(s) we wanted to answer?
  Is SHR associated with the in-hospital prognosis in non-surgical patients with type 2 diabetes and heart failure?

(iii) What did we find?
  Compared to the nadir range of SHR at 0.79 to 1.08, both high and low SHR at admission elicit elevated risks of cardiac, kidney, and infectious adverse events during hospitalization in non-surgical patients with type 2 diabetes and heart failure.

(iv) What are the implications of our findings?
  Clinicians should understand the inconsistency between the blood glucose at admission and HbA1c indicates poor prognosis during hospitalization and warrants additional monitoring.

**Supplementary Information:**

The online version contains supplementary material available at 10.1186/s12933-022-01728-w.

## Introduction

Stress hyperglycemia is a physiological response of blood glucose levels to stressful events or severe diseases through enhanced inflammatory or neuro-hormonal activation [1], typically reflecting the severity of the corresponding disease [1–3]. Type 2 diabetes, however, impairs glucose response to these stressful events. Estimating stress hyperglycemia is challenging until recent literature validated stress hyperglycemia ratio (SHR) reflecting ‘true stress hyperglycemia’ in hospitalization [4–12].

Among more than 12.1 million adults with heart failure in China, one in six was diagnosed with type 2 diabetes [13], eliciting worsened short-term and long-term prognoses [14, 15]. People with heart failure frequently experience readmissions due to acute decompensation or comorbidities [16]. These conditions cause stress that may raise blood glucose. Nevertheless, it remains unclear what the magnitude of blood glucose elevation means to patients with type 2 diabetes and heart failure. Given SHR that predicts the prognosis in people with type 2 diabetes and other cardiovascular diseases such as acute myocardial infarction (AMI), ischemic stroke, and acute coronary syndrome (ACS), we hypothesized that the SHR may also reflect the severity of the disease in patients with type 2 diabetes and heart failure [5–12]. This study thus investigates the association of different levels of SHR with the in-hospital cardiac, kidney, and infectious events in patients with type 2 diabetes and heart failure.

## Research design and methods

### Data source and study population

This cohort retrospectively extracted data of patients with diabetes hospitalized with heart failure from the electronic medical record (EMR)-based multicenter database of diabetes, namely West China Electronic medical record Collaboration Of DiabEtes (WECODe) [17]. WECODe captures longitudinal EMR data of patients with diabetes in both inpatient and outpatient settings from several tertiary hospitals in Sichuan Province, China, since January 2011 (Additional file 1: Appendix S1). The criteria for identifying people with diabetes in inpatient and outpatient settings from EMR were summarized in our previous paper, separately [17, 18]. WECODe links de-identified data from eight sources of EMR, demographic records, medical and discharge summaries, prescription records, surgery records, laboratory records, vital sign records, glucose monitoring records, and diagnosis records. The Big Data Platform at West China Hospital of Sichuan University (WCH-BDP) facilitates data storage and analysis [19].

Eligible adults with type 2 diabetes recorded in WECODe: (1) were discharged between January 1, 2011, and June 30, 2019; (2) had a diagnosis of “New York Heart Association (NYHA) class II, III, or IV” in the free text according to discharge diagnosis records; (3) stayed in hospital more than 2 days; and (4) had available records on diagnosis at discharge, prescription, blood glucose, and glycated hemoglobin A1c (HbA1c). Accounting for some data not missing at random, individuals were excluded if any of their key characteristics at admission were not available (serum creatinine, serum alanine aminotransferase [ALT], blood glucose, HbA1c, hemoglobin, low-density lipoprotein [LDL-c], N-terminal pro-B-type natriuretic peptide [NT-proBNP], and systolic blood pressure [BP]). Individuals were excluded if they had been admitted to, transferred to, or discharged from surgical departments.

If a patient was admitted to the hospital due to heart failure more than once, only the last record of hospitalization was assessed. The index date of each patient was the date of hospital admission. The observation period started 30 days before the index date and ended on the discharging day or 30 days after the index date. The baseline period started 30 days before the index date and ended two days after.

The ethics committee of West China Hospital, Sichuan University has approved this study (No. 2021–386; No. 2021–282; No. 2020–968). Patient consent was waived for this retrospective study of data from electronic medical records.

### Data collection and baseline characteristics

We retrieved and linked all prespecified medical data during both inpatient and outpatient settings within the observed period from WECODe. Additional file 1: Appendix S2 and Additional file 1: Tables S1, S2, S3 summarized the details for data linkage and methods to identify the baseline characteristics.

The estimated glomerular rate filtration (eGFR) was calculated according to the chronic kidney disease epidemiology collaboration (CKD-EPI) formula [20]. The status of impaired kidney function at baseline was identified as eGFR < 60 mL/min/1.73 m^2^ at baseline. The Charlson Comorbidity Index (CCI) was calculated to evaluate patient comorbidities based on the International Classification of Diseases 10th Revision (ICD-10) codes in the discharge diagnosis records [21].

### Exposure

Blood glucose at admission was identified as the first measurement on or next index date. Estimated average glucose levels were estimated by HbA1c using the formula, estimated average glucose level (mg/dl) = 28.7 × HbA1c (%) − 46.7 [22]. SHR is calculated as blood glucose at admission (mg/dl)/estimated average glucose level (mg/dl) [4]. We categorized SHR based on tertiles and set the second tertile as the reference.

### Follow-up and outcomes

We followed patients from the index date until a given adverse event occurred, they were discharged from the hospital, or until 30 days after the index date, whichever came first. The primary outcomes during hospitalization included composite cardiac events (the combination of death during hospitalization, requiring cardiopulmonary resuscitation, cardiogenic shock, and new episode of acute heart failure after admission), major acute kidney injury (AKI, defined as AKI stage 2 or 3), and major systemic infection (identified by the initiation of restricted antibiotics on the third calendar days after admission or later) during follow-up duration. The initiation of a given medication during hospitalization was defined as patients not receiving a given medication on admission and the following two calendar days, but a new given medication on the third calendar day after admission or later. The secondary outcomes included the separate adverse event of composite cardiac events, AKI at any stage, and initiating any antibiotics during follow-up. We defined requiring cardiopulmonary resuscitation as initiating an infusion of intravenous epinephrine, cardiogenic shock as prolonged hypotension (SBP ≤ 85 mmHg) with the presence of prescription of inotropic agents [23], new episode of acute heart failure after admission as initiating an infusion of intravenous morphine, and AKI at any stage following the previous paper [15, 24]. Antibiotics, including antibacterials and antimycotics, were identified according to Anatomical Therapeutic Chemical (ATC) Classification (https://www.whocc.no/atc_ddd_index/) (Additional file 1: Table S1). Only individuals without a given adverse event occurring on the index date and the following two calendar days were included for association analyses.

### Statistical analysis

#### Baseline characteristics

For continuous variables tested normally distributed by the Kolmogorov–Smirnov test (*P* ≥ 0.001), the paper presented them as mean ± standard deviation (SD) and compared them with a one-way analysis of covariance (ANOVA). For those not normally distributed, we presented them as median (25% percentile, 75% percentile) and compared them with the Kruskal–Wallis H test. Categorical variables, presented as frequencies (percentages), were compared using the Chi-square test. We performed Spearman`s correlation to investigate the association of SHR at admission with other baseline characteristics.

#### Non-linear association of SHR at admission with outcomes

We evaluated the non-linear association of SHR as a continuous exposure with a given outcome. First, we developed entropy balancing weights via weights optimization to achieve an exact balance of covariates moments [25], accounting for age, sex, baseline systolic BP, baseline eGFR, baseline NT-proBNP, admission department (Department of Cardiology /others), CCI, with or without ischemic heart disease at baseline, whether the use of insulin at baseline (Yes vs no), and whether the use of venous loop diuretics at baseline (Yes vs no). Additional file 1: Fig. S1 presented the correlation of SHR with each covariate before and after applying entropy balancing. For each outcome, a generalized logistic regression model with entropy balancing weights was used to obtain the odds ratio (OR) of each SHR (referent, SHR of 0.8) and fit the non-linear model. The corresponding 95% confidence intervals (CIs) were estimated using the percentile bootstrapping method [26, 27]. The SHR at admission was modeled with restricted cubic splines, and three knots located at the 25th, 50th, and 75th percentiles of SHR.

#### Comparing the risks of outcomes across SHR tertiles

We compared the risk of outcomes in the first and third tertiles against the second one. The analyses balanced each group using inverse probability weighting with entropy balancing with the absolute standardized mean differences (SMD) threshold of 0.10 (Additional file 1: Fig. S2). Generalized logistic regressions with entropy balancing weights were used to estimate the average treatment effect (ATE).

#### Subgroup analyses and sensitivity analyses

We conducted predefined subgroup analyses to assess the effect of SHR on the primary outcomes based on NYHA class (II or III vs IV), baseline HbA1c level (< 7.0% vs ≥ 7.0% [53.0 mmol/mol]), and impaired kidney function at baseline (eGFR < 60 mL/min/1.73 m^2^ vs eGFR ≥ 60 mL/min/1.73 m^2^). This study employed two sensitivity analyses to test the robustness of the study, by excluding patients with any episode of hypoglycemia during the baseline period and by excluding patients with a hyperglycemic crisis at baseline (see Additional file 1: Appendix S3).

All analyses were conducted using RStudio 2022.7.1.554 (R version 4.2.1). Statistical code for this analysis is freely accessible for any non-commercial reuse at: https://github.com/Yiling-Zhou/SHR_in-hospital-prognosis_HFandDiabetes. A two-sided *P* value < 0.05 was considered statistically significant.

## Results

### Baseline characteristics

**Fig. 1:**
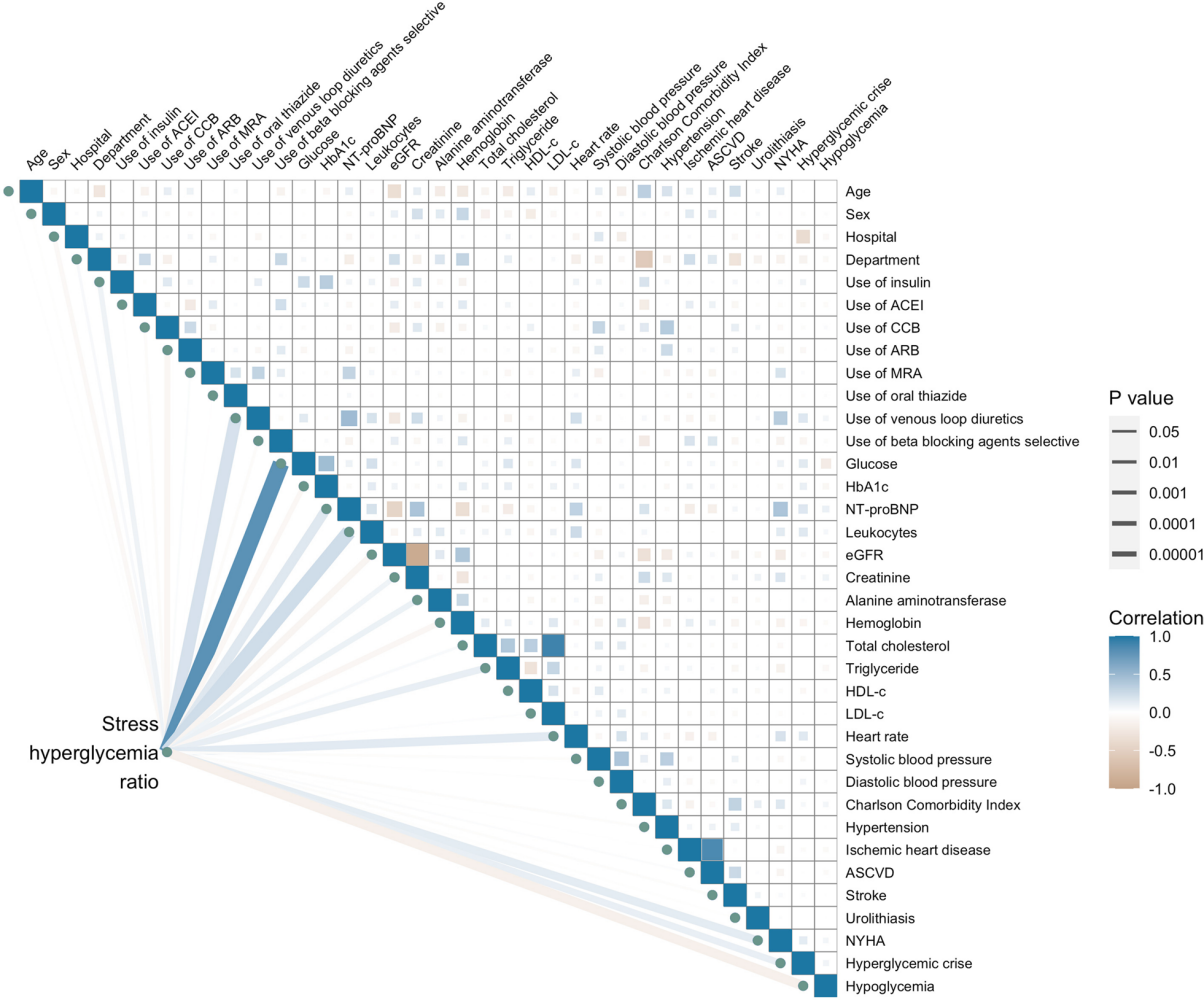
Correlation heatmap of baseline characteristics. *ACEI* angiotensin-converting enzyme inhibitor, *ARB* angiotensin II, receptor blockers*, CCB* calcium channel blocker, *MRA* aldosterone receptor antagonists, *HbA1c* glycated hemoglobin A1c, *NT-proBNP*, N-terminal pro-B-type natriuretic peptide, *eGFR* estimated glomerular filtration rate, *HDL-c* high-density lipoprotein, *LDL-c* low-density lipoprotein, *ASCVD* atherosclerotic cardiovascular disease, *SHR* stress hyperglycemia ratio, *NYHA* New York Heart Association. The color represents the Spearman correlation coefficient, (*r*s, will always take values from− 1 to 1). The brown color indicates a negative correlation, and the blue one indicates a positive correlation. The closer *r*s is to zero, the weaker the correlation between the two variables will be, and the lighter the color will be. The size of the square and the width of the line represent the significant level in statistics, constructed based on the transformation of Spearman`s P, − log_10_ (Spearman`s P), with the cutoff points, − log_10_ (0.00001), − log_10_ (0.0001), − log_10_ (0.001), − log_10_ (0.01), − log_10_ (0.05). The larger the size of the square is (the wider the line is), the smaller Spearman`s P is

This study included 2875 non-surgical patients with type 2 diabetes hospitalized with heart failure (Additional file 1: Fig. S3), with a median length of stay in hospital of 10 days (Interquartile Range, IQR, 5 to 17 days) (Additional file 1: Fig. S4). Table 1 shows their baseline characteristics. The median age was 71.2 years (IQR, 63.5 to 78.2 years). The percentage of females was 38.7%. The second tertile of SHR is located at 0.79 to 1.08. The percentage of patients experiencing a hyperglycemic crisis at baseline was highest in the third tertile (1st tertile, 2.3% vs 2nd tertile, 2.1% vs 3rd tertile, 8.6%). The portion of patients experiencing hypoglycemia at baseline was highest in the first tertile (1st tertile, 6.9% vs 2nd tertile, 0.3% vs 3rd tertile, 1.0%). The level of NT-proBNP was highest in the third tertile of SHR. The portions of insulin users, venous loop diuretics users, patients with impaired kidney function at baseline and patients diagnosed with NYHA class IV at baseline were all highest in the third tertile. Before applying entropy balancing, Fig. 1 illustrated the correlation map among baseline characteristics.

**Table 1:** Baseline characteristics of the total study population

Characteristics	Overall N = 2875	Stress hyperglycemia ratio			*P* value
		First tertile N = 927	Second tertile N = 972	Third tertile N = 976	
Primary variables					
Stress hyperglycemia ratio, range	0.16 to 4.45	0.16 to 0.78	0.79 to 1.08	1.09 to 4.45	
Stress hyperglycemia ratio	0.92 [0.73, 1.23]	0.67 [0.58, 0.73]	0.92 [0.85, 0.99]	1.40 [1.21, 1.64]	< 0.001
Blood glucose at admission, mmol/L	8.3 [6.3, 11.7]	5.8 [5.0, 6.9]	7.8 [6.8, 9.3]	12.8 [10.6, 16.6]	< 0.001
HbA1c, %	7.1 [6.4, 8.2]	7.2 [6.6, 8.5]	6.9 [6.3, 7.9]	7.1 [6.4, 8.3]	< 0.001
NYHA class					
Class II	1277 (44.4)	447 (48.2)	470 (48.4)	360 (36.9)	< 0.001
Class III	1036 (36.0)	338 (36.5)	351 (36.1)	347 (35.6)	
Class IV	562 (19.5)	142 (15.3)	151 (15.5)	269 (27.6)	
Demographics					
Age, years	71.2 [63.5, 78.2]	71.1 [63.0, 77.9]	71.5 [63.6, 78.3]	71.1 [64.0, 78.7]	0.53
Sex, female, n (%)	1113 (38.7)	355 (38.3)	371 (38.2)	387 (39.7)	0.76
Medical and discharge summaries					
Smoking, n (%)	1211 (42.1)	386 (41.6)	407 (41.9)	418 (42.8)	0.86
Stop smoking, n (%)	843 (29.3)	279 (30.1)	289 (29.8)	275 (28.2)	0.61
Drinking, n (%)	814 (28.3)	256 (27.6)	272 (28.0)	286 (29.3)	0.69
Stop drinking, n (%)	144 (5.0)	57 (6.1)	43 (4.4)	44 (4.5)	0.16
Admission department, cardiology, n (%)	1716 (59.7)	518 (55.9)	605 (62.2)	593 (60.8)	0.01
Laboratory test					
HbA1c ≥ 7.0%, n (%)	1584 (55.1)	546 (58.9)	483 (49.7)	555 (56.9)	< 0.001
NT-proBNP, pg/mL	937.0 [231.0, 3652.5]	809.0 [211.5, 3291.0]	633.0 [171.5, 2671.5]	1487.0 [368.0, 4912.0]	< 0.001
Leukocytes, 10*9/L	6.77 [5.47, 8.74]	6.62 [5.42, 8.16]	6.54 [5.33, 8.04]	7.38 [5.73, 10.23]	< 0.001
Serum creatinine, μmol/L	92.9 [75.0, 123.0]	91.0 [74.0, 122.0]	90.0 [73.1, 114.0]	97.9 [77.3, 135.0]	< 0.001
ALT, U/L	21.0 [14.0, 33.0]	21.0 [14.0, 32.0]	21.0 [14.0, 31.0]	22.0 [14.0, 38.0]	0.01
Hemoglobin, g/L	126.0 [109.0, 140.0]	127.0 [110.0, 140.0]	129.0 [114.0, 142.0]	122.0 [104.0, 137.0]	< 0.001
eGFR < 60 mL/(min*1.73m^2^), n (%)	1268 (44.1)	388 (41.9)	385 (39.6)	495 (50.7)	< 0.001
eGFR, mL/(min*1.73m^2^)	64.90 [45.41, 84.11]	67.43 [46.25, 84.75]	67.93 [49.52, 84.67]	59.35 [40.01, 82.54]	< 0.001
TC, mmol/L	3.74 [3.09, 4.55]	3.69 [3.06, 4.50]	3.73 [3.08, 4.57]	3.78 [3.16, 4.61]	0.21
TG, mmol/L	1.35 [0.97, 1.98]	1.24 [0.94, 1.74]	1.37 [0.99, 2.04]	1.50 [1.04, 2.22]	< 0.001
HDL-c, mmol/L	1.10 [0.89, 1.37]	1.12 [0.89, 1.37]	1.11 [0.90, 1.36]	1.09 [0.88, 1.37]	0.42
LDL-c, mmol/L	1.98 [1.48, 2.64]	1.96 [1.48, 2.64]	1.95 [1.48, 2.64]	2.02 [1.49, 2.62]	0.90
Vital signs					
Heart rate, beats/minute	80 [70, 90]	78 [68, 87]	78 [68, 89]	82 [72, 98]	< 0.001
Systolic blood pressure, mmHg	132 [118, 148]	132 [118, 150]	131 [119, 148]	132 [118, 149]	0.78
Diastolic blood pressure, mmHg	75 [67, 84]	74.0 [67, 84]	75.0 [67, 82]	75 [67, 84]	0.46
Comorbidity					
Charlson Comorbidity Index	3 [2, 8]	4 [2, 8]	3 [2, 7]	4 [2, 8]	0.01
Hypoglycemia, n (%)	77 (2.7)	64 (6.9)	3 (0.3)	10 (1.0)	< 0.001
Hyperglycemic crises, n (%)	125 (4.3)	21 (2.3)	20 (2.1)	84 (8.6)	< 0.001
Hypertension, n (%)	2095 (72.9)	681 (73.5)	722 (74.3)	692 (70.9)	0.22
IHD, n (%)	2085 (72.5)	670 (72.3)	702 (72.2)	713 (73.1)	0.90
ASCVD, n (%)	2253 (78.4)	718 (77.5)	755 (77.7)	780 (79.9)	0.35
Stroke, n (%)	578 (20.1)	204 (22.0)	191 (19.7)	183 (18.8)	0.19
Urolithiasis, n (%)	93 (3.2)	32 (3.5)	36 (3.7)	25 (2.6)	0.33
Medication use					
Use of insulin, n (%)	967 (33.6)	295 (31.8)	273 (28.1)	399 (40.9)	< 0.001
Use of ACEI, n (%)	422 (14.7)	128 (13.8)	140 (14.4)	154 (15.8)	0.46
Use of CCB, n (%)	802 (27.9)	273 (29.4)	274 (28.2)	255 (26.1)	0.26
Use of ARB, n (%)	704 (24.5)	246 (26.5)	267 (27.5)	191 (19.6)	< 0.001
Use of MRA, n (%)	659 (22.9)	198 (21.4)	229 (23.6)	232 (23.8)	0.39
Use of oral thiazide, n (%)	94 (3.3)	26 (2.8)	35 (3.6)	33 (3.4)	0.60
Use of venous loop diuretics, n (%)	685 (23.8)	168 (18.1)	177 (18.2)	340 (34.8)	< 0.001
Use of beta blocking agents selective, n (%)	1033 (35.9)	339 (36.6)	374 (38.5)	320 (32.8)	0.03

*NYHA* New york heart association, *NT-proBNP* N-terminal pro-B-type natriuretic peptide, *eGFR* estimated glomerular filtration rate, *ALT*, alanine aminotransferase, *LDL-c* low-density lipoprotein, *TC* total cholesterol, *HDL-c* high-density lipoprotein, *TG* triglyceride, *HbA1c* glycated hemoglobin A1c, *IHD* ischemic heart disease, *ASCVD* atherosclerotic cardiovascular disease, *ACEI* angiotensin-converting enzyme inhibitor, *ARB* angiotensin II, receptor blockers, *CCB* calcium channel blocker, *MRA* aldosterone receptor antagonists

### Adverse events during hospitalization across people with different SHR

#### Primary outcomes

**Fig. 2:**
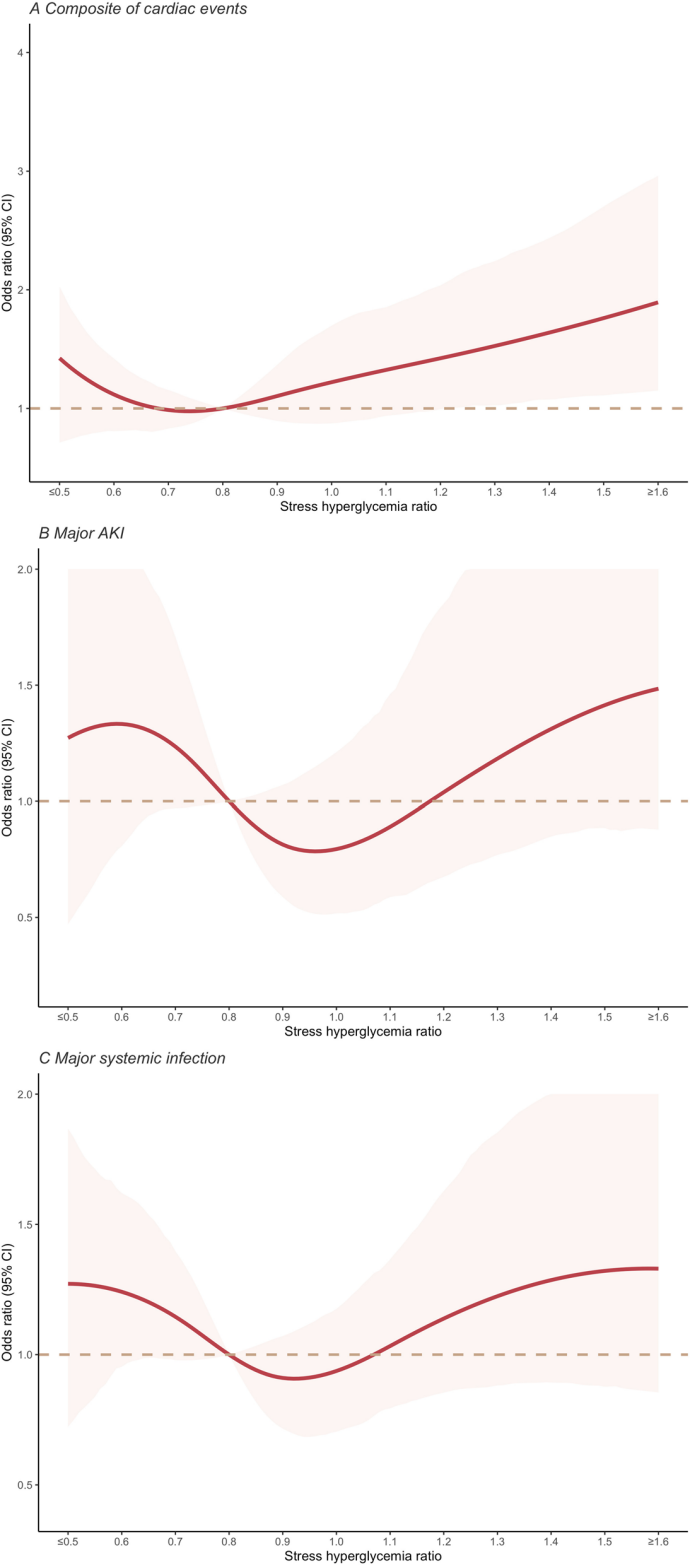
Nonlinear association of stress hyperglycemia ratio with primary outcomes in the total study population *AKI* acute kidney injury. A, Stress hyperglycemia ratio and composite of cardiac events; B, Stress hyperglycemia ratio and major acute kidney injury; C, Stress hyperglycemia ratio and major systemic infection. All analyses were adjusted for age, sex, baseline systolic blood pressure, baseline estimated glomerular filtration rate, baseline N-terminal pro-B-type natriuretic peptide, admission department (Department of Cardiology /others), Charlson Comorbidity Index, with or without ischemic heart disease at baseline, whether use of insulin at baseline (Yes vs no), and whether use of venous loop diuretics at baseline (Yes vs no).

**Fig. 3:**
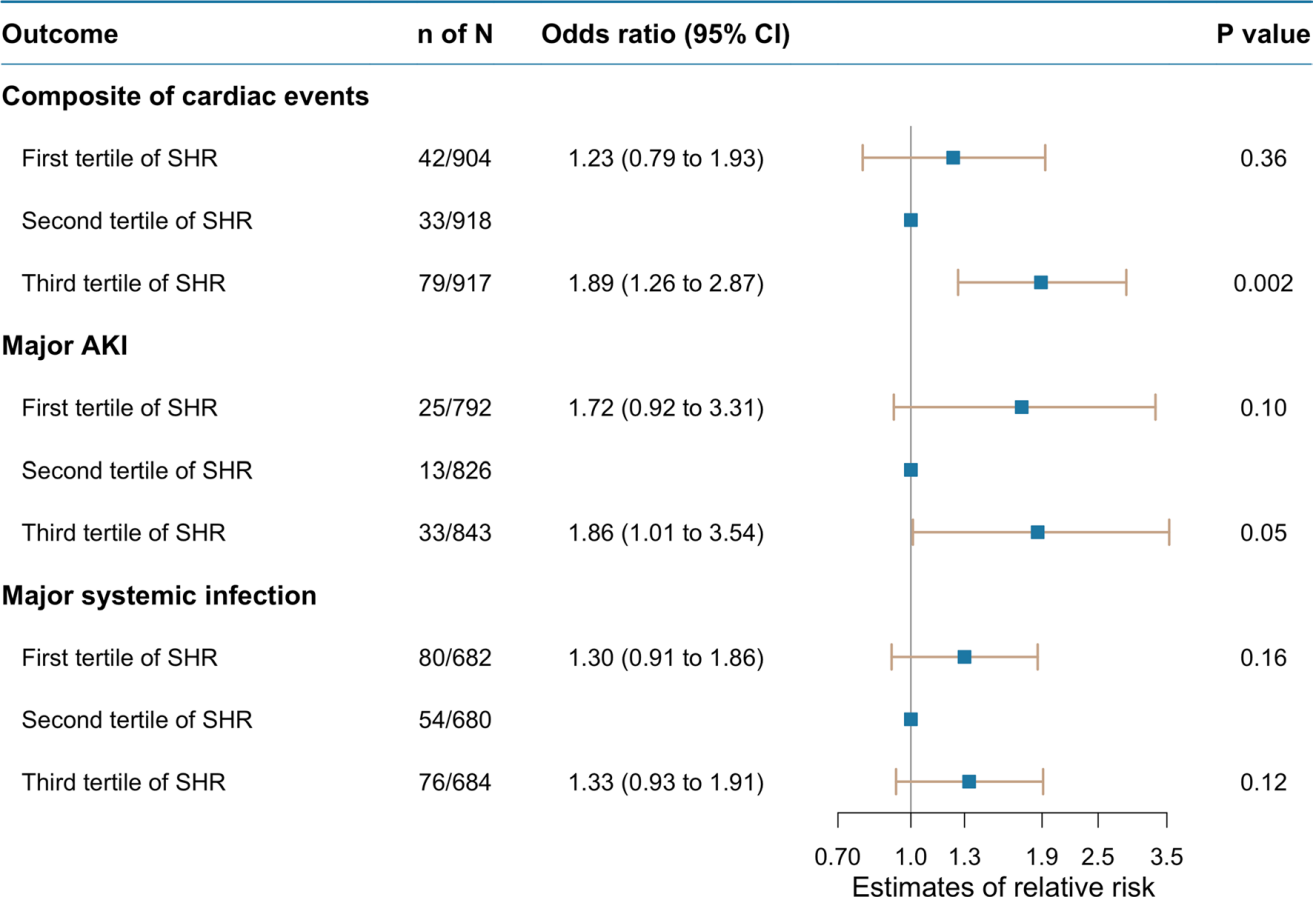
The adjusted odds ratio of stress hyperglycemia ratio tertiles for primary outcomes in the total study population, *CI* confidence interval, *SHR* stress hyperglycemia ratio. All analyses were adjusted for age, sex, baseline systolic blood pressure, baseline estimated glomerular filtration rate, baseline N-terminal pro-B-type natriuretic peptide, admission department (Dpartment of Cardiology/others), Charlson Comorbidity Index, with or without ischemic heart disease at baseline, whether use of insulin at baseline (Yes vs no), and whether use of venous loop diuretics at baseline (Yes vs no)

The analysis for composite cardiac events included 2739 patients with 154 events. Figure 2 showed a U-shaped association between the SHR and composite cardiac events, with a nadir at an SHR of 0.78. Compared with patients with the SHR in the second tertile, the adjusted OR for composite cardiac events was 1.89 (95% CI 1.26 to 2.87, *P* = 0.002) for patients with a higher SHR (the third tertile), and 1.23 (95% CI 0.79 to 1.93, *P* = 0.36) for patients with a lower SHR (the first tertile) (Fig. 3). Subgroup analyses did not identify any subgroup effects (Additional file 1: Fig. S5, S6).

The analysis for major AKI included 2461 patients with 71 events and indicated a U-shaped association between the SHR and the outcome with a nadir at an SHR of 0.96 (Fig. 2). Compared with patients with the SHR in the second tertile, the adjusted OR for major AKI was 1.86 (95% CI 1.01 to 3.54, *P* = 0.05) for patients with a higher SHR, and 1.72 (95% CI 0.92 to 3.31, *P* = 0.10) for patients with a lower SHR (Fig. 3). Subgroup analysis indicated whether having impaired kidney function at baseline affected this association. When patients with impaired kidney function at baseline, the risk of this outcome was significantly higher in both patients with a lower SHR and those with a higher SHR (the first tertile, OR, 2.60, 95% CI 1.17 to 6.31, *P* = 0.03; the third tertile, OR, 3.01, 95% CI 1.39 to 7.19, *P* = 0.01), but not in those with a better kidney function at baseline (Additional file 1: Fig. S5, S6).

The analysis for major systemic infection included 2046 patients with 210 events. SHR had a U-shaped association with major systemic infection, with a nadir at an SHR of 0.92 (Fig. 2). Compared with patients with an SHR in the second tertile, the adjusted OR for experiencing major systemic infection during hospitalization was 1.33 (95% CI 0.93 to 1.91, *P* = 0.12) for patients with a higher SHR, and 1.30 (95% CI 0.91 to 1.86, *P* = 0.16) for patients with a lower SHR (Fig. 3). When patients with impaired kidney function at baseline, both the lower and higher SHR were significantly associated with a higher risk of this outcome (the first tertile, OR 1.92, 95% CI 1.15 to 3.29, *P* = 0.01; the third tertile, OR, 1.78, 95% CI 1.05 to 3.05, *P* = 0.03), but not when patients with a better kidney function at baseline (Additional file 1: Fig. S5, S6). Both sensitivity analyses confirmed the robustness of the results (Additional file 1: Fig. S9, S10, S11, S12).

#### Secondary outcomes

After applying entropy balancing, details of the nonlinear association of SHR with secondary outcomes were illustrated in Additional file 1: Fig. S7. Compared with patients with an SHR in the second tertile, the adjusted OR for death during hospitalization was 3.07 (95% CI, 1.70 to 5.87, *P* < 0.001) for patients with a higher SHR. The adjusted OR for requiring cardiopulmonary resuscitation during hospitalization was 2.24 (95% CI, 1.26 to 4.14, *P* = 0.008) for patients with a higher SHR. The adjusted OR for AKI at any stage during hospitalization was 1.59 (95% CI 1.10 to 2.30, *P* = 0.01) for patients with a higher SHR (Additional file 1: Fig. S8).

## Discussion

This study showed a U-shaped association between SHR and in-hospital cardiac, kidney, and infectious adverse events in non-surgical hospitalized patients with type 2 diabetes and heart failure. For patients with impaired kidney function, both high SHR and low SHR elicit elevated risks of major AKI and major systemic infection during hospitalization.

Our study, based on existing evidence from observational studies regarding SHR as a meaningful measure for stress hyperglycemia in patients with AMI, ACS, stroke, trauma [28], or COVID-19 [29], for the first time assessed how SHR signifies in non-surgical patients hospitalized with type 2 diabetes and heart failure. This large study population comes from a multi-center database, increasing the generalizability of our findings to Chinese people with type 2 diabetes hospitalized with heart failure. Adopting inverse probability weighting to control for prespecified confounders allows us to obtain the ATE of SHR on the outcomes for the underlying population. The nonlinear and tertile analyses confirm each other, yielding robust evidence to support the qualification of SHR as a biomarker for stress hyperglycemia, as well as breaking the impasse where existing studies showed inconsistent associations of stress hyperglycemia, estimated by blood glucose at admission, with in-hospital prognosis in people with type 2 diabetes and heart failure.

Our findings are in line with previous observations in hospitalized patients with type 2 diabetes and other cardiovascular diseases [4–7, 11], providing support for the prognostic value of SHR. Our study also exhibits U-shaped associations of SHR with in-hospital adverse events in people with type 2 diabetes hospitalized with heart failure, suggesting the nadir range of SHR at 0.79 to 1.08. This is consistent with a recent study that SHR is nonlinearly associated with in-hospital cardiac death and MI in patients with ACS and the inflection point is at SHR of 0.78 [9]. A prior study in Spain [8] found the third tertile of SHR with a mean of around 1.2 was associated with the lowest 4 year mortality in people with diabetes hospitalized for acute heart failure, which was in line with our findings to some extent. However, this Spain study did not provide the range of each SHR tertile and it only recruited 570 people living with type 2 diabetes.

Our results highlight the importance of estimating stress hyperglycemia to anticipate the prognosis of people living with type 2 diabetes hospitalized for heart failure. Using this information, clinicians should pay more attention to those with low and high SHR estimates and take action to prevent cardiac, kidney, and infectious events. Calculating SHR at a population level, policymakers and public health practitioners may anticipate the burden of hospitalized patients with type 2 diabetes and heart failure in different settings. Automatic calculation of SHR in the EMR, in these scenarios, facilitates such a process. Previous studies located the ‘ideal’ range for SHR within 0.75 to 1.68 depending on the outcome, which was consistent with our findings. To be noted, although 1.0 reflects the consistency of blood glucose at home and at the hospital, a relatively lower glucose level at admission may anticipate a better prognosis, especially for cardiac events, most likely owing to people receiving regular glucose-lowering medications before or during their stay.

The physiological mechanism underlying stress hyperglycemia and short-term prognosis remains not well established. Elevated blood glucose at admission marks the severity of the acute condition and disease leading to hospitalization, enhanced by subsequent inflammatory and neuroendocrine derangements (1). This hyperglycemia, in turn, worsens cardiovascular outcomes and reduces renal perfusion by aggravating oxidative stress response, inflammatory state, endothelial dysfunction, volume depletion, and dehydration (1). In contrast, some people in severe conditions may experience hypoglycemia, the mechanism of which is far from well understood. The fallen blood glucose maybe results from improper insulin or oral drug use, long fasting or digestive impairment, impaired counter-regulation partial adrenal insufficiency, all of which pushed people into poor prognoses [30–32].

### Limitations

Several limitations merit discussion. Firstly, EMR captures clinical routine data in both outpatient and inpatient settings, lack of long-term follow-up. Considering this limitation, we only analyzed data derived from the last hospitalization of patients and focused on the in-hospital adverse events. Secondly, the lack of hormone measurements in our dataset hampered our understanding of the poor prognosis in patients with lower SHR. We hypothesize that relative hypocortisolism is one of the underlying physiological explanations, which needs to be confirmed by further studies. Thirdly, although the Chinese FDA approved the first SGLT2 inhibitor in 2017, the class of drugs are unavailable in the studied scenario until late 2019. Analysis may be necessary. Finally, like many other studies investigating stressful hyperglycemia [4, 7, 9], since many participants were admitted to the hospital from the emergency room, this study was unable to measure the impact of diet on glucose. It calls for prospective studies to collect information regarding diet intake, intravenous glucose infusion and blood glucose measure.

## Conclusion

In summary, both high and low SHR indicate poor prognosis during hospitalization in non-surgical patients with heart failure and type 2 diabetes, especially in those with impaired kidney function at admission.

## Supplementary Information

**Additional file 1: Appendix S1.** Brief summary of WECODe. **Appendix S2.** Details of data collection. **Appendix S3.** The definition of a hyperglycemic crisis. **Appendix S4.** WECODe Study Group. **Table S1.** The details of prescription information. **Table S2**. The details of laboratory tests. **Table S3.** Identification of comorbidities from discharge diagnosis records using the International Classification of Diseases, 10th Revision (ICD-10) codes, or free text. **Table S4.** Details of stress hyperglycemia ratio tertiles for each outcome in the total study population and each subgroup. **Fig S1.** Correlations of stress hyperglycemia ratio with covariates before and after applying entropy balancing. **Figure S2.** Absolute mean difference of covariates across stress hyperglycemia ratio tertiles before and after applying entropy balancing. **Figure S3.** The flowchart of the selection of the study population. **Figure S4.** The length of stay in hospital across stress hyperglycemia ratio tertiles. **Figure S5.** Subgroup analyses of the non-linear association between stress hyperglycemia ratio and primary outcomes during hospitalization. **Figure S6.** Subgroup analyses of the association between stress hyperglycemia ratio tertiles and primary outcomes in patients with heart failure and diabetes. **Figure S7**. Nonlinear association of stress hyperglycemia ratio with secondary outcomes in the total study population**. Figure S8.** The adjusted odds ratio of stress hyperglycemia ratio tertiles for secondary outcomes in the total study population. **Figure S9.** Nonlinear association of stress hyperglycemia ratio with primary outcomes in the total study population in a sensitivity analysis by excluding patients with hypoglycemia at baseline. **Figure S10.** The adjusted odds ratio of stress hyperglycemia ratio tertiles for primary outcomes in the total study population by excluding patients with hypoglycemia at baseline. **Figure S11.** Nonlinear association of stress hyperglycemia ratio with primary outcomes in the total study population in a sensitivity analysis by excluding patients with hyperglycemic crises at baseline. **Figure S12.** The adjusted odds ratio of stress hyperglycemia ratio tertiles for primary outcomes in the total study population by excluding patients with hyperglycemic crises at baseline.

## Data Availability

The datasets generated from electronic medical records during the current study are not publicly available for reasons of patient privacy.
