# Supplementary material for: 'Stress hyperglycemia ratio and in-hospital prognosis in non-surgical patients with heart failure and type 2 diabetes
Source: Cardiovasc Diabetol. 2022 Dec 26;21:290. doi: 10.1186/s12933-022-01728-w (PMC9791974; doi:10.1186/s12933-022-01728-w)
Supplement: Supplementary file 1 — Additional file 1: Appendix S1. Brief summary of WECODe. Appendix S2. Details of data collection. Appendix S3. The definition of a hyperglycemic crisis. Appendix S4. WECODe Study Group. Table S1. The details of prescription information. Table S2. The details of laboratory tests. Table S3. Identification of comorbidities from discharge diagnosis records using the International Classification of Diseases, 10th Revision (ICD-10) codes, or free text. Table S4. Details of stress hyperglycemia ratio tertiles for each outcome in the total study population and each subgroup. Fig S1. Correlations of stress hyperglycemia ratio with covariates before and after applying entropy balancing. Figure S2. Absolute mean difference of covariates across stress hyperglycemia ratio tertiles before and after applying entropy balancing. Figure S3. The flowchart of the selection of the study population. Figure S4. The length of stay in hospital across stress hyperglycemia ratio tertiles. Figure S5. Subgroup analyses of the non-linear association between stress hyperglycemia ratio and primary outcomes during hospitalization. Figure S6. Subgroup analyses of the association between stress hyperglycemia ratio tertiles and primary outcomes in patients with heart failure and diabetes. Figure S7. Nonlinear association of stress hyperglycemia ratio with secondary outcomes in the total study population. Figure S8. The adjusted odds ratio of stress hyperglycemia ratio tertiles for secondary outcomes in the total study population. Figure S9. Nonlinear association of stress hyperglycemia ratio with primary outcomes in the total study population in a sensitivity analysis by excluding patients with hypoglycemia at baseline. Figure S10. The adjusted odds ratio of stress hyperglycemia ratio tertiles for primary outcomes in the total study population by excluding patients with hypoglycemia at baseline. Figure S11. Nonlinear association of stress hyperglycemia ratio with primary outcomes in the total study po [file 12933_2022_1728_MOESM1_ESM.docx]

**Supplementary Material**

Contents

[List of appendices	2](#_Toc119015400)

[Appendix A: Brief summary of WECODe	2](#_Toc119015401)

[Appendix B: Details of data collection	2](#_Toc119015402)

[Appendix C: The definition of a hyperglycemic crisis	3](#_Toc119015403)

[Appendix D: WECODe Study Group	4](#_Toc119015404)

[Supplementary Figures and Table	5](#_Toc119015405)

[Supplementary Tables	5](#_Toc119015406)

[Supplementary Table S1: The details of prescription information	5](#_Toc119015407)

[Supplementary Table S2: The details of laboratory tests	6](#_Toc119015408)

[Supplementary Table S3: Identification of comorbidities from discharge diagnosis records using the International Classification of Diseases, 10th Revision (ICD-10) codes, or free text	6](#_Toc119015409)

[Supplementary Table S4: Details of stress hyperglycemia ratio tertiles for each outcome in total study population and each subgroup	8](#_Toc119015410)

[Supplementary Figures	11](#_Toc119015411)

[Supplementary Figure S1: Correlations of stress hyperglycemia ratio with covariates before and after applying entropy balancing	11](#_Toc119015412)

[Supplementary Figure S2: Absolute mean difference of covariates across stress hyperglycemia ratio tertiles before and after applying entropy balancing	12](#_Toc119015413)

[Supplementary Figure S3: The flowchart of the selection of study population	13](#_Toc119015414)

[Supplementary Figure S4: The length of stay in hospital across stress hyperglycemia ratio tertiles	14](#_Toc119015415)

[Supplementary Figure S5: Subgroup analyses of non-linear association between stress hyperglycemia ratio and primary outcomes during hospitalization	15](#_Toc119015416)

[Supplementary Figure S6: Subgroup analyses of the association between stress hyperglycemia ratio tertiles and primary outcomes in patients with heart failure and diabetes	20](#_Toc119015417)

[Supplementary Figure S7: Nonlinear association of stress hyperglycemia ratio with secondary outcomes in the total study population	23](#_Toc119015418)

[Supplementary Figure S8: The adjusted odds ratio of stress hyperglycemia ratio tertiles for secondary outcomes in the total study population	27](#_Toc119015419)

[Supplementary Figure S9: Nonlinear association of stress hyperglycemia ratio with primary outcomes in the total study population in sensitivity analysis by excluding patients with hypoglycemia at baseline	28](#_Toc119015420)

[Supplementary Figure S10: The adjusted odds ratio of stress hyperglycemia ratio tertiles for primary outcomes in the total study population by excluding patients with hypoglycemia at baseline	31](#_Toc119015421)

[Supplementary Figure S11: Nonlinear association of stress hyperglycemia ratio with primary outcomes in the total study population in sensitivity analysis by excluding patients with hyperglycemic crises at baseline	32](#_Toc119015422)

[Supplementary Figure S12: The adjusted odds ratio of stress hyperglycemia ratio tertiles for primary outcomes in the total study population by excluding patients with hyperglycemic crises at baseline	35](#_Toc119015423)

# List of appendices

## Appendix A: Brief summary of WECODe

The electronic medical record (EMR)-based multicenter database of diabetes, namely West China Electronic medical record Collaboration Of DiabEtes (WECODe), captures longitudinal EMR data of patients with diabetes in both inpatient and outpatient settings from hospitals in Sichuan Province, China, since January 2011. WECODe includes inpatients if they (1) attended the inpatient department with a discharge diagnosis according to International Classification of Diseases 10th Revision (ICD-10), including codes E10 to E14, fasting glucose > 7.0 mmol/L, 2-hour blood glucose after 75 g glucose challenge > 11.1 mmol/L, random glucose > 11.1 mmol/L, or glycated hemoglobin A1c (HbA1c) > 6.5% (48 mmol/mol); (2) were ≥ 18 years old; and (3) were Chinese; recruits outpatients if they attended the outpatient department and had a diagnosis of “diabetes” in the free text or ICD-10 codes including E10 to E14 in the EMR. It links anonymized data from eight sources of EMR, demographic records, medical and discharge summaries, prescription records, surgery records, laboratory records, vital sign records, glucose monitoring records, and diagnosis records. All data are archived in the big data platform at West China Hospital of Sichuan University. Up to now, the database, as an ongoing work, has already obtained data from five hospitals, and covered > 491,350 people with diabetes, 193,683 people in the inpatient setting with a median follow-up of 43 days, and 297,667 people in the outpatient setting with a median follow-up of 3.2 years.

## Appendix B: Details of data collection

We retrieved and linked all prespecified medical data during both inpatient and outpatient settings within the observed period from WECODe, namely age, sex, smoking status, alcohol consumption, discharge diagnosis with ICD-10 codes and free text, admission department (Department of Cardiology/others), admission date, discharge date, discharge department and patient status on the discharge; the dates and records of medication prescription (**Supplementary Table S1**); vital signs (systolic and diastolic blood pressure [BP], and heart rate); laboratory test results (**Supplementary Table S2**). **Supplementary Table S3** summarized the ICD-10 codes or diagnosis with free text for other comorbidities.

We only evaluated the last hospitalization with a discharge diagnosis of “New York Heart Association class Ⅱ, Ⅲ, or Ⅳ” if patients were admitted more than once; the admission date was set as the index date for each eligible individual; the observed period was from 3 months before index date to the discharge date or 30 days after the index date, whichever came first; baseline window was set as the period of 30 days prior and two days post-index date. We used the record of a given parameter closest to the index date during the baseline window as the baseline value. Especially, the baseline HbA1c was identified as the closest measurement to the index date 90 days before and two days after the index date; the baseline heart rate, systolic BP, diastolic BP, and prescriptions were identified as the first records on or next index date.

## Appendix C: The definition of a hyperglycemic crisis

The criteria of a hyperglycemic crisis included patients at baseline with 1) any blood glucose ≥ 30mmol/L; 2) serum sodium > 145 mmol/L and any blood glucose ≥ 16.7 mmol/L; 3) any blood glucose ≥ 13.9mmol/L and any of the following: PH <7.3, beta-hydroxybutyric acid ≥ 1 mmol/L, or urine ketone ≥ 2+; or 4) were diagnosed with diabetic ketoacidosis (DKA) or hyperglycemic hyperosmolar state (HHS) (Supplementary Table S3).

## Appendix D: WECODe Study Group

Participating investigators in the three hospitals involved in this study, who are in the WECODe Study Group

**Full name** (Affiliation)

**Tao Zheng** (Department of Informatics, West China Hospital, Sichuan University, Chengdu, 610041, China)

**Hao Yang** (Department of Informatics, West China Hospital, Sichuan University, Chengdu, 610041, China)

**Tianyi Yang** (Department of Informatics, West China Hospital, Sichuan University, Chengdu, 610041, China)

**Ran Liu** (Department of Informatics, West China Hospital, Sichuan University, Chengdu, 610041, China)

**Shuangqing Li** (Department of General Medicine, West China Hospital, Sichuan University, Chengdu, 610041, China)

**Kang An** (Department of General Medicine, West China Hospital, Sichuan University, Chengdu, 610041, China)

**Zhenmei An** (Department of Endocrinology and Metabolism, West China Hospital, Sichuan University, Chengdu, 610041, China)

**Jing Li** (Department of Endocrinology and Metabolism, West China Hospital, Sichuan University, Chengdu, 610041, China)

**Zhilong Mei** (Department of Endocrinology and Metabolism, Second people's Hospital of Ya'an City, Ya'an, 625000, China)

**Hao Chen** (Department of Informatics, Second people's Hospital of Ya'an City, Ya'an, 625000, China)

**Min Gan** (Department of Nutrition, Second people's Hospital of Ya'an City, Ya'an, 625000, China)

**Wanrong Feng** (Medical Records Department, Second people's Hospital of Ya'an City, Ya'an, 625000, China)

**Pengxu Chen** (Department of Informatics, The First People's Hospital of Shuangliu District, Chengdu, 610200, China)

**Yi Chen** (Department of Informatics, The First People's Hospital of Shuangliu District, Chengdu, 610200, China)

# Supplementary Figures and Table

## Supplementary Tables

### Supplementary Table S1: The details of prescription information

| Medication | Anatomical Therapeutic Chemical (ATC) Classification |
| --- | --- |
| Insulin | Insulins and analogs for injection, fast-acting;  Insulins and analogs for injection, intermediate-acting;  Insulins and analogs for injection, intermediate- or long-acting combined with fast-acting;  Insulins and analogs for injection, long-acting. |
| ACEI | ACE inhibitors, plain;  ACE inhibitors and diuretics;  ACE inhibitors and dihydropyridine derivatives;  ACE inhibitors, plain and folic acid and derivatives;  ACE inhibitors, other combinations. |
| ARB | ARB, plain;  ARB and diuretics;  ARB and CCB;  ARB, other combinations. |
| CCB | Dihydropyridine derivatives;  Dihydropyridine derivatives and ACE inhibitors, plain. |
| MRA | Aldosterone antagonists |
| Thiazide | Thiazides, plain |
| Venous loop diuretics | Furosemide;  Bumetanide;  Torasemide. |
| Beta blocking agents selective | Beta blocking agents selective. |
| Morphine | ATC code: N02AA01 |
| Epinephrine | ATC code: C01CA24 |
| Milrinone | ATC code: C01CE02 |
| Dobutamine | ATC code: C01CA07 |
| Dopamine | ATC code: C01CA04 |
| Norepinephrine | ATC code: C01CA03 |
| Antibacterials | |
| All | Antibacterials for systemic use;  Antibacterials for systemic use, anti-inflammatory, and antirheumatic products;  Antibacterials for systemic use, antigout preparations;  Antibacterials for systemic use, antibacterials for systemic use. |
| Restricted or special | ATC codes including J01AA02; J01AA05; J01AA08; J01CA09; J01CA10 and J01CG01; J01CA16; J01CF05 and J01CA04; J01CR02; J01CR03; J01CR05; J01DB07; J01DB12; J01DC01; J01DC07; J01DC09; J01DC12; J01DD01; J01DD02; J01DD06; J01DD07; J01DD10; J01DD11; J01DD12; J01DD13; J01DD15; J01DD16; J01DD18; J01DD52; J01DD62; J01DD63; J01DE01; J01DE02; J01DF01; J01DH02; J01DH03; J01DH51; J01DH55; J01DI03; J01FA10; J01FA13; J01GB; J01GB01; J01GB03; J01MA; J01MA01; J01MA02; J01MA06; J01MA07; J01MA08; J01MA09; J01MA10; J01MA14; J01MA16; J01MA18; J01MA22; J01XA; J01XA01; J01XA02; J01XB02; J01XX08. |
| Antimycotics | |
| All | Antimycotics for systemic use. |
| Restricted or special | ATC codes including J02AA01; J02AC02; J02AC03; J02AX04; J02AX05. |

Abbreviations: ACEI, angiotensin-converting enzyme inhibitor; ARB, angiotensin II, receptor blockers; CCB, calcium channel blocker; MRA, aldosterone receptor antagonists.

### Supplementary Table S2: The details of laboratory tests

| Laboratory tests | | | |
| --- | --- | --- | --- |
| - Low-density lipoprotein  - Total cholesterol  - High-density lipoprotein  - Triglyceride | - Alanine aminotransferase  - Hemoglobin  - N-terminal pro-B-type natriuretic peptide  - Serum creatinine  - Leukocytes | - Blood glucose  - Glycated hemoglobin A1c  - Fasting glucose  - 2-hour blood glucose after - 75 g glucose challenge  - Random glucose | PH  Serum sodium  Beta hydroxybutyric acid  Urine ketones |

### Supplementary Table S3: Identification of comorbidities from discharge diagnosis records using the International Classification of Diseases, 10th Revision (ICD-10) codes, or free text

| Comorbidities | ICD-10 codes | Free text |
| --- | --- | --- |
| Hypertension | I10 to I15 | Free text relating to hypertension; but excluding free text relating to without hypertension, or with or without a family history of hypertension |
| Ischemic heart disease (IHD) | I20 to I25 | Free text describing heart problems caused by narrowed heart arteries, such as myocardial infarction, angina, IHD, or coronary artery stenosis; but excluding free text relating to without IHD, or with or without a family history of IHD |
| Stroke | I61, I63, I64 | Free text describing brain ischemia or brain hemorrhage; but excluding free text presenting without stroke |
| Atherosclerotic cardiovascular disease (ASCVD) | I20 to I25, I61, I63, I64 | This study only evaluates the most frequently occurring diseases of ASCVD, free text describing IHD, cerebrovascular disease; but excludes free text showing without ASCVD |
| Urolithiasis | N20.001 to N20.005, N20.101, N20.102, N20.201 to N20.203, N20.901, N20.902, N21.001, N21.002, N21.101, N23.01, N23.02 | Free text describing the formation of stones (calculi) in the kidney, bladder, or urethra; excluding free text showing without urolithiasis |
| Diabetic ketoacidosis (DKA) or hyperglycemic hyperosmolar state (HHS) | E11.0, E11.1, E12.0, E12.1,  E13.0, E13.1,  E14.0, E14.1, | Chinese free text relating to DKA or HHS |

### Supplementary Table S4: Details of stress hyperglycemia ratio tertiles for each outcome in the total study population and each subgroup

| Population | Stress hyperglycemia ratio | | |
| --- | --- | --- | --- |
|  | **First tertile** | **Second tertile** | **Third tertile** |
| Composite of cardiac events | | | |
| Total | 0.16 ~ 0.78 [904] | 0.79 ~ 1.07 [918] | 1.08 ~ 4.38 [917] |
| NYHA |  |  |  |
| NYHA class 2 or 3 | 0.16 ~ 0.77 [742] | 0.78 ~ 1.03 [741] | 1.04 ~ 4.38 [757] |
| NYHA class 4 | 0.19 ~ 0.84 [165] | 0.85 ~ 1.23 [167] | 1.24 ~ 3.71 [167] |
| HbA1c at admission |  |  |  |
| HbA1c <7.0% | 0.16 ~ 0.80 [406] | 0.81 ~ 1.05 [418] | 1.06 ~ 3.71 [414] |
| HbA1c ≥7.0% | 0.24 ~ 0.76 [493] | 0.77 ~ 1.09 [507] | 1.10 ~ 4.38 [501] |
| eGFR at admission |  |  |  |
| <60 mL/min/1.73 m^2^ | 0.19 ~ 0.79 [394] | 0.80 ~ 1.13 [396] | 1.14 ~ 4.38 [401] |
| ≥60 mL/min/1.73 m^2^ | 0.16 ~ 0.77 [501] | 0.78 ~ 1.02 [522] | 1.03 ~ 3.46 [525] |
| Major acute kidney injury | | | |
| Total | 0.16 ~ 0.77 [792] | 0.78 ~ 1.04 [826] | 1.05 ~ 4.38 [843] |
| NYHA |  |  |  |
| NYHA class 2 or 3 | 0.16 ~ 0.77 [683] | 0.78 ~ 1.02 [678] | 1.03 ~ 4.38 [708] |
| NYHA class 4 | 0.19 ~ 0.81 [128] | 0.82 ~ 1.15 [129] | 1.16 ~ 3.71 [135] |
| HbA1c at admission |  |  |  |
| HbA1c <7.0% | 0.16 ~ 0.79 [348] | 0.80 ~ 1.03 [373] | 1.04 ~ 3.71 [361] |
| HbA1c ≥7.0% | 0.25 ~ 0.76 [456] | 0.77 ~ 1.06 [456] | 1.07 ~ 4.38 [467] |
| eGFR at admission |  |  |  |
| <60 mL/min/1.73 m^2^ | 0.19 ~ 0.78 [294] | 0.79 ~ 1.08 [309] | 1.09 ~ 4.38 [309] |
| ≥60 mL/min/1.73 m^2^ | 0.16 ~ 0.77 [505] | 0.78 ~ 1.02 [524] | 1.03 ~ 3.27 [520] |
| Major systemic infection | | | |
| Total | 0.16 ~ 0.77 [682] | 0.78 ~ 1.02 [680] | 1.03 ~ 2.96 [684] |
| NYHA |  |  |  |
| NYHA class 2 or 3 | 0.16 ~ 0.76 [577] | 0.77 ~ 1.01 [630] | 1.02 ~ 2.96 [608] |
| NYHA class 4 | 0.20 ~ 0.77 [76] | 0.78 ~ 1.12 [76] | 1.13 ~ 2.84 [79] |
| HbA1c at admission |  |  |  |
| HbA1c <7.0% | 0.16 ~ 0.78 [297] | 0.79 ~ 1.00 [315] | 1.01 ~ 2.84 [316] |
| HbA1c ≥7.0% | 0.27 ~ 0.75 [365] | 0.76 ~ 1.04 [379] | 1.05 ~ 2.96 [374] |
| eGFR at admission |  |  |  |
| <60 mL/min/1.73 m^2^ | 0.27 ~ 0.77 [266] | 0.78 ~ 1.06 [265] | 1.07 ~ 2.96 [269] |
| ≥60 mL/min/1.73 m^2^ | 0.16 ~ 0.76 [398] | 0.77 ~ 0.99 [417] | 1.00 ~ 2.77 [431] |
| Death during hospitalization | | | |
| Total | 0.16 ~ 0.78 [927] | 0.79 ~ 1.08 [972] | 1.09 ~ 4.45 [976] |
| NYHA |  |  |  |
| NYHA class 2 or 3 | 0.16 ~ 0.77 [755] | 0.78 ~ 1.04 [777] | 1.05 ~ 4.45 [781] |
| NYHA class 4 | 0.19 ~ 0.85 [183] | 0.86 ~ 1.25 [188] | 1.26 ~ 3.71 [191] |
| HbA1c at admission |  |  |  |
| HbA1c <7.0% | 0.16 ~ 0.80 [415] | 0.81 ~ 1.07 [444] | 1.08 ~ 4.45 [432] |
| HbA1c ≥7.0% | 0.24 ~ 0.76 [506] | 0.77 ~ 1.10 [542] | 1.11 ~ 4.38 [536] |
| eGFR at admission |  |  |  |
| <60 mL/min/1.73 m^2^ | 0.19 ~ 0.80 [411] | 0.81 ~ 1.15 [426] | 1.16 ~ 4.45 [431] |
| ≥60 mL/min/1.73 m^2^ | 0.16 ~ 0.77 [515] | 0.78 ~ 1.03 [550] | 1.04 ~ 3.46 [542] |
| Requiring cardiopulmonary resuscitation | | | |
| Total | 0.16 ~ 0.78 [923] | 0.79 ~ 1.08 [970] | 1.09 ~ 4.45 [966] |
| NYHA |  |  |  |
| NYHA class 2 or 3 | 0.16 ~ 0.77 [754] | 0.78 ~ 1.04 [776] | 1.05 ~ 4.45 [780] |
| NYHA class 4 | 0.19 ~ 0.85 [180] | 0.86 ~ 1.25 [183] | 1.26 ~ 3.71 [186] |
| HbA1c at admission |  |  |  |
| HbA1c <7.0% | 0.16 ~ 0.80 [414] | 0.81 ~ 1.06 [435] | 1.07 ~ 4.45 [436] |
| HbA1c ≥7.0% | 0.24 ~ 0.76 [503] | 0.77 ~ 1.10 [540] | 1.11 ~ 4.38 [531] |
| eGFR at admission |  |  |  |
| <60 mL/min/1.73 m^2^ | 0.19 ~ 0.80 [409] | 0.81 ~ 1.15 [422] | 1.16 ~ 4.45 [425] |
| ≥60 mL/min/1.73 m^2^ | 0.16 ~ 0.77 [513] | 0.78 ~ 1.03 [550] | 1.04 ~ 3.46 [540] |
| New episode of acute heart failure after admission | | | |
| Total | 0.16 ~ 0.78 [912] | 0.79 ~ 1.07 [925] | 1.08 ~ 4.38 [941] |
| NYHA |  |  |  |
| NYHA class 2 or 3 | 0.16 ~ 0.77 [746] | 0.78 ~ 1.03 [742] | 1.04 ~ 4.38 [768] |
| NYHA class 4 | 0.19 ~ 0.84 [169] | 0.85 ~ 1.23 [179] | 1.24 ~ 3.71 [174] |
| HbA1c at admission |  |  |  |
| HbA1c <7.0% | 0.16 ~ 0.80 [408] | 0.81 ~ 1.05 [420] | 1.06 ~ 3.71 [423] |
| HbA1c ≥7.0% | 0.24 ~ 0.76 [499] | 0.77 ~ 1.09 [512] | 1.10 ~ 4.38 [516] |
| eGFR at admission |  |  |  |
| <60 mL/min/1.73 m^2^ | 0.19 ~ 0.80 [404] | 0.81 ~ 1.14 [404] | 1.15 ~ 4.38 [407] |
| ≥60 mL/min/1.73 m^2^ | 0.16 ~ 0.77 [507] | 0.78 ~ 1.02 [524] | 1.03 ~ 3.46 [532] |
| Cardiogenic shock | | | |
| Total | 0.16 ~ 0.78 [922] | 0.79 ~ 1.08 [964] | 1.09 ~ 4.45 [950] |
| NYHA |  |  |  |
| NYHA class 2 or 3 | 0.16 ~ 0.77 [752] | 0.78 ~ 1.04 [775] | 1.05 ~ 4.45 [769] |
| NYHA class 4 | 0.19 ~ 0.84 [174] | 0.85 ~ 1.25 [184] | 1.26 ~ 3.71 [182] |
| HbA1c at admission |  |  |  |
| HbA1c <7.0% | 0.16 ~ 0.80 [414] | 0.81 ~ 1.06 [434] | 1.07 ~ 4.45 [427] |
| HbA1c ≥7.0% | 0.24 ~ 0.76 [502] | 0.77 ~ 1.10 [536] | 1.11 ~ 4.38 [523] |
| eGFR at admission |  |  |  |
| <60 mL/min/1.73 m^2^ | 0.19 ~ 0.80 [410] | 0.81 ~ 1.15 [418] | 1.16 ~ 4.45 [415] |
| ≥60 mL/min/1.73 m^2^ | 0.16 ~ 0.77 [511] | 0.78 ~ 1.03 [548] | 1.04 ~ 3.46 [534] |
| Acute kidney injury at any stage | | | |
| Total | 0.16 ~ 0.77 [792] | 0.78 ~ 1.04 [826] | 1.05 ~ 4.38 [843] |
| NYHA |  |  |  |
| NYHA class 2 or 3 | 0.16 ~ 0.77 [683] | 0.78 ~ 1.02 [678] | 1.03 ~ 4.38 [708] |
| NYHA class 4 | 0.19 ~ 0.81 [128] | 0.82 ~ 1.15 [129] | 1.16 ~ 3.71 [135] |
| HbA1c at admission |  |  |  |
| HbA1c <7.0% | 0.16 ~ 0.79 [348] | 0.80 ~ 1.03 [373] | 1.04 ~ 3.71 [361] |
| HbA1c ≥7.0% | 0.25 ~ 0.76 [456] | 0.77 ~ 1.06 [456] | 1.07 ~ 4.38 [467] |
| eGFR at admission |  |  |  |
| <60 mL/min/1.73 m^2^ | 0.19 ~ 0.78 [294] | 0.79 ~ 1.08 [309] | 1.09 ~ 4.38 [309] |
| ≥60 mL/min/1.73 m^2^ | 0.16 ~ 0.77 [505] | 0.78 ~ 1.02 [524] | 1.03 ~ 3.27 [520] |
| Initiating any antibiotics | | | |
| Total | 0.16 ~ 0.76 [626] | 0.77 ~ 1.01 [654] | 1.02 ~ 2.84 [657] |
| NYHA |  |  |  |
| NYHA class 2 or 3 | 0.16 ~ 0.76 [559] | 0.77 ~ 1.00 [585] | 1.01 ~ 2.77 [587] |
| NYHA class 4 | 0.20 ~ 0.76 [67] | 0.77 ~ 1.09 [70] | 1.10 ~ 2.84 [69] |
| HbA1c at admission |  |  |  |
| HbA1c <7.0% | 0.16 ~ 0.78 [286] | 0.79 ~ 1.00 [300] | 1.01 ~ 2.84 [301] |
| HbA1c ≥7.0% | 0.27 ~ 0.75 [350] | 0.76 ~ 1.02 [346] | 1.03 ~ 2.77 [354] |
| eGFR at admission |  |  |  |
| <60 mL/min/1.73 m^2^ | 0.27 ~ 0.76 [243] | 0.77 ~ 1.05 [261] | 1.06 ~ 2.84 [254] |
| ≥60 mL/min/1.73 m^2^ | 0.16 ~ 0.76 [383] | 0.77 ~ 0.99 [393] | 1.00 ~ 2.77 [403] |

Abbreviations: NYHA, New York Heart Association; HbA1c, glycated hemoglobin A1c; eGFR, estimated glomerular rate filtration.

HbA1c level at admission of 7.0% is equal to 53.0 mmol/mol.

Patients with impaired kidney function at baseline were identified by eGFR at baseline <60 mL/min/1.73 m^2^.

## Supplementary Figures

### Supplementary Figure S1: Correlations of stress hyperglycemia ratio with covariates before and after applying entropy balancing

Abbreviations: Department, admission department (Cardiology vs others); IHD, ischemic heart disease; CCI, Charlson Comorbidity Index; NT-proBNP, N-terminal pro-B-type natriuretic peptide; BP, blood pressure; eGFR, estimated glomerular rate filtration.

A, composite of cardiac events; B, major acute kidney injury; C, major systemic infection.

### Supplementary Figure S2: Absolute mean difference of covariates across stress hyperglycemia ratio tertiles before and after applying entropy balancing

Abbreviations: Department, admission department (Cardiology vs others); IHD, ischemic heart disease; CCI, Charlson Comorbidity Index; NT-proBNP, N-terminal pro-B-type natriuretic peptide; BP, blood pressure; eGFR, estimated glomerular rate filtration.

A, composite of cardiac events; B, major acute kidney injury; C, major systemic infection.

### Supplementary Figure S3: The flowchart of the selection of the study population

**5,483** adults with type 2 diabetes and NYHA class Ⅱ, Ⅲ, or Ⅳ having records of HbA1c and glucose at admission*

(01/01/2011~30/06/2019)

**Excluded**

**-** **2,518**: Without baseline key variables^†^

**2,965** individuals with complete baseline key variables^†^

**2,875** individuals not in surgical departments

**Excluded**

**- 90**: Admitted to, transferred to, or discharged from surgical departments

**Composite of cardiac events**

**2,739** patients

**Major systemic infection**

**2,046** patients

**Excluded**

- Individuals having a given adverse event on the index day or the following two calendar days

**Major AKI**

**2,461** patients

*Adults with type 2 diabetes were diagnosed with NYHA class Ⅱ, Ⅲ, or Ⅳ at admission or during hospitalization, with available records on diagnosis at discharge, prescription, and blood glucose and glycated hemoglobin A1c at admission, covered by West China Electronic medical record Collaboration Of DiabEtes between 01/01/2011 to 30/06/2019.

^†^Baseline key characteristics include serum creatinine, serum alanine aminotransferase, hemoglobin, low-density lipoprotein, blood glucose, glycated hemoglobin A1c, N-terminal pro-B-type natriuretic peptide, and systolic blood pressure.

NYHA, New York Heart Association; AKI, Acute kidney injury; HbA1c, glycated hemoglobin A1c.

### Supplementary Figure S4: The length of stay in hospital across stress hyperglycemia ratio tertiles

### Supplementary Figure S5: Subgroup analyses of the non-linear association between stress hyperglycemia ratio and primary outcomes during hospitalization

Abbreviations: HbA1c, glycated hemoglobin A1c; NYHA, New York Heart Association; AKI, acute kidney injury; eGFR, estimated glomerular rate filtration.

A, composite of cardiac events; B, major acute kidney injury; C, major systemic infection.

All analyses were adjusted for age, sex, baseline systolic blood pressure, baseline estimated glomerular filtration rate, baseline N-terminal pro-B-type natriuretic peptide, admission department (Department of Cardiology /others), Charlson Comorbidity Index, with or without ischemic heart disease at baseline, whether use of insulin at baseline (Yes vs no), and whether use of venous loop diuretics at baseline (Yes vs no). HbA1c level at admission of 7.0% is equal to 53.0 mmol/mol.

Patients with impaired kidney function at baseline were identified by eGFR at baseline <60 mL/min/1.73 m^2^.

### Supplementary Figure S6: Subgroup analyses of the association between stress hyperglycemia ratio tertiles and primary outcomes in patients with heart failure and diabetes

(1) **Composite of cardiac events**

**(2) Major AKI**

**(3) Major systemic infection**

Abbreviations: SHR, stress hyperglycemia ratio; HbA1c, glycated hemoglobin A1c; AKI, acute kidney injury; eGFR, estimated glomerular rate filtration; CI, confidence interval; NYHA, New York Heart Association.

All analyses were adjusted for age, sex, baseline systolic blood pressure, baseline estimated glomerular filtration rate, baseline N-terminal pro-B-type natriuretic peptide, admission department (Department of Cardiology /others), Charlson Comorbidity Index, with or without ischemic heart disease at baseline, whether use of insulin at baseline (Yes vs no), and whether use of venous loop diuretics at baseline (Yes vs no). HbA1c level at admission of 7.0% is equal to 53.0 mmol/mol.

Patients with impaired kidney function at baseline were identified by eGFR at baseline <60 mL/min/1.73 m^2^.

### Supplementary Figure S7: Nonlinear association of stress hyperglycemia ratio with secondary outcomes in the total study population

Abbreviations: HbA1c, glycated hemoglobin A1c; NYHA, New York Heart Association; AKI, acute kidney injury; AHF, acute heart failure.

A, SHR and death; B, SHR and cardiopulmonary resuscitation; C, SHR and new episode of AHF; D, SHR and cardiogenic shock; E, SHR and AKI at any stage; F, SHR and initiating any antibiotics.

All analyses were adjusted for age, sex, baseline systolic blood pressure, baseline estimated glomerular filtration rate, baseline N-terminal pro-B-type natriuretic peptide, admission department (Department of Cardiology /others), Charlson Comorbidity Index, with or without ischemic heart disease at baseline, whether use of insulin at baseline (Yes vs no), and whether use of venous loop diuretics at baseline (Yes vs no).

### Supplementary Figure S8: The adjusted odds ratio of stress hyperglycemia ratio tertiles for secondary outcomes in the total study population

Abbreviations: CI, confidence interval; SHR, stress hyperglycemia ratio.

All analyses were adjusted for age, sex, baseline systolic blood pressure, baseline estimated glomerular filtration rate, baseline N-terminal pro-B-type natriuretic peptide, admission department (Department of Cardiology /others), Charlson Comorbidity Index, with or without ischemic heart disease at baseline, whether use of insulin at baseline (Yes vs no), and whether use of venous loop diuretics at baseline (Yes vs no).

### Supplementary Figure S9: Nonlinear association of stress hyperglycemia ratio with primary outcomes in the total study population in a sensitivity analysis by excluding patients with hypoglycemia at baseline

Abbreviations: AKI, acute kidney injury.

A, Stress hyperglycemia ratio and composite of cardiac events; B, Stress hyperglycemia ratio and major acute kidney injury; C, Stress hyperglycemia ratio and major systemic infection.

All analyses were adjusted for age, sex, baseline systolic blood pressure, baseline estimated glomerular filtration rate, baseline N-terminal pro-B-type natriuretic peptide, admission department (Department of Cardiology /others), Charlson Comorbidity Index, with or without ischemic heart disease at baseline, whether use of insulin at baseline (Yes vs no), and whether use of venous loop diuretics at baseline (Yes vs no).

### Supplementary Figure S10: The adjusted odds ratio of stress hyperglycemia ratio tertiles for primary outcomes in the total study population by excluding patients with hypoglycemia at baseline

Abbreviations: CI, confidence interval; SHR, stress hyperglycemia ratio.

All analyses were adjusted for age, sex, baseline systolic blood pressure, baseline estimated glomerular filtration rate, baseline N-terminal pro-B-type natriuretic peptide, admission department (Department of Cardiology /others), Charlson Comorbidity Index, with or without ischemic heart disease at baseline, whether use of insulin at baseline (Yes vs no), and whether use of venous loop diuretics at baseline (Yes vs no).

### Supplementary Figure S11: Nonlinear association of stress hyperglycemia ratio with primary outcomes in the total study population in a sensitivity analysis by excluding patients with hyperglycemic crises at baseline

Abbreviations: AKI, acute kidney injury.

A, Stress hyperglycemia ratio and composite of cardiac events; B, Stress hyperglycemia ratio and major acute kidney injury; C, Stress hyperglycemia ratio and major systemic infection.

All analyses were adjusted for age, sex, baseline systolic blood pressure, baseline estimated glomerular filtration rate, baseline N-terminal pro-B-type natriuretic peptide, admission department (Department of Cardiology /others), Charlson Comorbidity Index, with or without ischemic heart disease at baseline, whether use of insulin at baseline (Yes vs no), and whether use of venous loop diuretics at baseline (Yes vs no).

### Supplementary Figure S12: The adjusted odds ratio of stress hyperglycemia ratio tertiles for primary outcomes in the total study population by excluding patients with hyperglycemic crises at baseline

Abbreviations: CI, confidence interval; SHR, stress hyperglycemia ratio.

All analyses were adjusted for age, sex, baseline systolic blood pressure, baseline estimated glomerular filtration rate, baseline N-terminal pro-B-type natriuretic peptide, admission department (Department of Cardiology /others), Charlson Comorbidity Index, with or without ischemic heart disease at baseline, whether use of insulin at baseline (Yes vs no), and whether use of venous loop diuretics at baseline (Yes vs no).
